# Letter to the Editor regarding “Association between sedentary behavior and sleep quality among urban white-collar workers with or at risk of metabolic syndrome: a secondary analysis of a randomized 3-month workplace lifestyle intervention trial”

**DOI:** 10.1093/joccuh/uiag008

**Published:** 2026-02-20

**Authors:** Hao Shi, Kaiyang Xu, Chen Zhao, Yonggang Zhu

**Affiliations:** Department of Neurological Rehabilitation, The First Affiliated Hospital of Kangda College of Nanjing Medical University/The First People's Hospital of Lianyungang, Lianyungang, Jiangsu, China; School of Acupuncture-Moxibustion and Tuina, Shanghai University of Traditional Chinese Medicine, Shanghai, China; School of Acupuncture-Moxibustion and Tuina, Shanghai University of Traditional Chinese Medicine, Shanghai, China; Department of Neurological Rehabilitation, The First Affiliated Hospital of Kangda College of Nanjing Medical University/The First People's Hospital of Lianyungang, Lianyungang, Jiangsu, China

**Keywords:** sedentary behavior, sleep quality, metabolic syndrome, white-collar workers, occupational health

Dear Editor,

We read with great interest the article entitled “Association between sedentary behavior and sleep quality among urban white-collar workers with or at risk of metabolic syndrome: a secondary analysis of a randomized 3-month workplace lifestyle intervention trial” by Ishikawa et al, regarding the within-person associations between objectively measured sedentary time and sleep quality in urban white-collar workers with or at risk of metabolic syndrome. The use of multilevel modeling to disaggregate between-person and within-person effects provides the granular evidence necessary for day-level workplace interventions. To further enhance the translational impact of these findings without increasing participant burden, we propose 3 targeted methodological additions.^1^

First, although the study focused on total daily sedentary time, exploring the accumulation pattern of sedentary behavior (SB)—characterized by prolonged sedentary bouts versus fragmented accumulation—could provide more nuanced insights.^2^ Although wrist-worn devices have known limitations in distinguishing physical sitting from standing, the temporal distribution of sedentary bouts (defined by the study’s <1.5 MET threshold) remains a viable proxy for behavioral phenotypes. For instance, research suggests that the health risks associated with SB may be more pronounced when sedentary time is accumulated in long, sustained sedentary bouts.^3^ Utilizing the intraday minute-by-minute data already captured by Fitbit Versa, future analyses could compute pattern measures like the Gini index or fragmentation index to determine whether the prolonged accumulation of low-MET periods specifically predicts poorer sleep quality.

Second, the relatively small effect size observed suggests that the relationship may not be strictly linear. We recommend exploring potential nonlinear thresholds or “tipping points” where SB begins to significantly impair sleep. Furthermore, given that sedentary behavior and physical activity are codependent within a 24-hour cycle, employing isotemporal substitution models—as demonstrated in recent multicenter studies^4^—could clarify the specific impact of reallocating sedentary time to physical activity. Considering that mental stress significantly impacts both activity patterns and sleep, incorporating perceived stress as a time-varying moderator could better capture how workplace pressure influences these daily associations.^5^

Third, we suggest situating these findings within a bidirectional feedback framework. Although the current model correctly orders day-level behavior to next-morning sleep, contemporary evidence suggests a vicious cycle where poor sleep independently increases next-day sedentary propensity. Reporting the reverse-direction model from sleep on Day t to sedentary behavior on Day t + 1 would clarify whether the observed associations are part of a self-reinforcing loop, which is vital for designing sustainable lifestyle interventions in populations at risk of metabolic syndrome.

We congratulate the authors on this robust contribution to occupational health. Addressing these nuances in sedentary phenotypes and bidirectionality would further elevate the clarity and practical relevance of the study for the *Journal of Occupational Health* readership.

Sincerely,

Hao Shi
